# Altering Fish Behavior by Sensing Swarm Patterns of Fish in an Artificial Aquatic Environment Using an Interactive Robotic Fish

**DOI:** 10.3390/s23031550

**Published:** 2023-01-31

**Authors:** Udaka A. Manawadu, Malsha De Zoysa, J. D. H. S. Perera, I. U. Hettiarachchi, Stephen G. Lambacher, Chinthaka Premachandra, P. Ravindra S. De Silva

**Affiliations:** 1 Graduate School of Computer Science and Engineering, University of Aizu, Fukushima 965-0006, Japan; 2 Centre of Robotics and Intelligent Systems, University of Sri Jayewardenepura, Nugegoda 10250, Sri Lanka; 3 School of Social Informatics, Aoyama Gakuin University, Tokyo 150-8366, Japan; 4 Graduate School of Engineering and Science, Shibaura Institute of Technology, Tokyo 135-8548, Japan

**Keywords:** underwater robotics, robotic fish, ostraciiform fish, motion detection, tracking, fish image processing

## Abstract

Numerous studies have been conducted to prove the calming and stress-reducing effects on humans of visiting aquatic environments. As a result, many institutions have utilized fish to provide entertainment and treat patients. The most common issue in this approach is controlling the movement of fish to facilitate human interaction. This study proposed an interactive robot, a robotic fish, to alter fish swarm behaviors by performing an effective, unobstructed, yet necessary, defined set of actions to enhance human interaction. The approach incorporated a minimalistic but futuristic physical design of the robotic fish with cameras and infrared (IR) sensors, and developed a fish-detecting and swarm pattern-recognizing algorithm. The fish-detecting algorithm was implemented using background subtraction and moving average algorithms with an accuracy of 78%, while the swarm pattern detection implemented with a Convolutional Neural Network (CNN) resulted in a 77.32% accuracy rate. By effectively controlling the behavior and swimming patterns of fish through the smooth movements of the robotic fish, we evaluated the success through repeated trials. Feedback from a randomly selected unbiased group of subjects revealed that the robotic fish improved human interaction with fish by using the proposed set of maneuvers and behavior.

## 1. Introduction

People tend to work under stress due to the workload exceeding human cognitive limits. They often engage in various activities to relax and calm themselves [1]. Spending time with fish kept for decorative purposes is one of the most popular activities to promote stress-reducing and calming effects [2]. Fish are beautiful creatures of mother nature. Due to their magnificent swimming behaviors, people enjoy spending their leisure time with fish, inspiring them to carry out inventions, the most obvious being related to transportation and the most recent being robotic fish [3]. Considering the therapeutic inclinations of fish, medical institutions have installed fish tanks to calm stressed patients and as a pre-treatment for anxiety, fear, frustration, and depression [4,5,6].

Spending time with ornamental fish is one of the most popular time-pass activities for stress reduction, it has also been revealed that observing fish at an aquarium reduces stress, lowers blood pressure effectively, and provides numerous health benefits [2,7,8,9]. While observing fish can provide stress-reducing benefits, it is important to note that fish may not always be active or perform attractive maneuvers. In these situations, people may not be able to fully engage with the aquatic environment and may not be able to achieve the full calming effects. To ensure that people receive maximum benefits, there needs to be a way to make the aquarium environment more interactive, even when fish are not active. Keeping a person on-hand to do this is not practical, so there is a need for an agent or system that can make the environment more active and engaging when people are present. Additionally, this agent should be able to monitor fish swarm behaviors and ensure that the fish are living in good conditions.

Robotic fish can serve as automated agents in various endeavors to enhance human–fish interaction. At present, a significant portion of robotic fish research is dedicated to creating biomimetic robots, monitoring ocean conditions, and analyzing fish behavior [10,11,12,13]. Additionally, they can be utilized in aquaculture to monitor fish behavior and optimize fish farming conditions. Some robotic fish also feature cameras, sensors, and other apparatus to gather data on water quality, temperature, and other parameters.

Previous studies have not utilized a robotic fish to enhance human interaction and guide the alteration of fish swarm patterns. Robotic technologies can be used not only for biomimicry but also to enhance human–fish interaction. This research aims to explore the suitable design for a robotic fish, the behavior of fish in response to the robot’s behavior, and methods for recognizing fish swarm patterns. Therefore, the key contributions of this research are:

- Designing a futuristic and minimalistic robotic fish with an Ostraciiform tail that can operate in a wide range of water environments.
- Analyzing fish behaviors and fish swarm patterns using the robotic fish and recognizing these patterns using a proposed machine learning algorithm.
- Performing fish-food drop activity based on the identified fish swarm patterns and analyzing the resulting changes in fish behavior.
- Collecting user feedback about the robot fish and its behavior.

## 2. Literature Review

Over the past two decades, significant research has been conducted on the social effects of fish on humans. For example, a study by Deborah et al. found that aquarium settings with higher-level fish species were linked to greater decreases in heart rate [8]. Kidd et al.’s study concludes that interacting with fish has a significant impact on patients with mental disorders [14]. Gee et al.’s study suggests that fish movements are primarily responsible for the calming effects of watching them, as their weightless and otherworldly movements immediately reduce tension [15]. Many studies have attempted to modify fish behavior in artificial aquatic settings through techniques such as lighting and decorative items. The most substantial research indicates that feeding fish can directly affect their behavior and activity [16].

Underwater robotics is a rapidly growing field with a wide range of applications. It is already being used for various tasks such as object identification, vessel hull inspections, and underwater survey missions [10,11,12]. The locomotion of fish has inspired researchers to design robots to mimic the motion and dynamics of natural fish, namely robotic fish, which is an Autonomous Underwater Vehicle (AUV) designed to obtain fish-like swimming behaviors [3,17,18]. Robotuna was the first-ever robotic fish prototype in the world, designed and developed by MIT in 1994. Since then, underwater robotics has reached new heights in achieving biomimicry in robotic fish [19]. A study by Wei Zhao et al. conducted using a Proportional Integral Derivative (PID) control algorithm, which controls the depth at which the fish was operated using sonar, three infrared (IR) sensors, and one wireless duplex communication module, marks a milestone in underwater robotics [20]. The research mainly focused on enhancing swimming performance and achieving smooth gait transitions of the robot fish. A free-locomotive robotic fish propelled by Ionic Polymer Metal Composites (IPMCs) was highlighted in a study by Matteo et al. [21]. K. H. Low et al. designed a robotic fish fin that can mimic the fin locomotion capability of a natural fish [22]. Kopman et al. developed a heading control algorithm for the robotic fish propelled by the tail undulation [23]. A world-first bionic underwater fish drone (BIKI) utilizes Ostraciiform fish-like swimming developed and based on a business model that brings more powerful features to the spotlight [24]. The setbacks that were prominent in earlier designs, e.g., being noisy, unsafe, the requirement of wire for moving, short battery life, and high production and maintenance costs, were significantly lowered by BIKI. Cazenille et al. designed a biomimetic robotic fish that has similar behavior to a zebrafish and showed that the robot can be integrated into a group of zebrafish, mimic their behavior, and exhibit similar collective dynamics compared to fish-only groups [25].

The area of underwater object detection and tracking has been vastly improving. However, despite prior studies, this area is still searching for perfection. Zivkovic et al. proposed a method for background subtraction with an improved adaptive Gaussian mixture model [26]. A study by Yi et al. proposed a background subtraction technique and a temporal difference that requires minimal computational complexity yet yield higher accuracy than the prevailing approaches [27]. Detecting fish underwater is challenging due to varying external factors: fluid vortexes significantly affect the camera’s vibration, resulting in impaired quality video, and disturbances from aquatic plants, decorative items, and rocks add to the challenge [28,29]. Tracking fish is more challenging with a dynamic camera, which has drawn the minds of researchers and resulted in numerous studies on detecting, counting, and monitoring fish underwater using different methods and applications [30]. Spampinato et al. proposed using the Continuously Adaptive Mean Shift (CamShift) algorithm to detect and count fish in the water, giving significant results in low contrast and unconstrained environments [31]. A method to study fish behaviors and estimate fish trajectories by overcoming underwater environmental changes was proposed by Morais et al. [32].

The use of robotic fish is mostly in the areas of exploration and inspection of underwater life [33,34]. Altering fish swarm patterns to manipulate the fish behavior in order to increase human interaction is rather a newer area of research. Biomimicry in robotic fish has been vastly improved while lesser attention has been given to the use of a robotic fish to change the behavior of fish to improve human interaction [35,36]. In addition to the use of robotic fish in the exploration and examination of aquatic life, another benefit of robotic fish is to improve human interaction with fish. In order to increase the interaction of humans with fish, the behavior of the fish can be altered.

The literature review revealed a significant amount of research on creating robots that mimic fish behaviors and identifying fish underwater, as well as the impact of fish on the human mind. However, we found that no previous studies have been conducted on recognizing fish swarm patterns or using robotics to enhance human–fish interaction. Therefore, there is a need to develop a robot that can actively change and track the swarm patterns of fish, in order to make the aquatic environment more dynamic. This study proposes a robotic fish to keep track of the swarm behavior of fish and manipulate that behavior by performing an effective and necessary pre-defined set of actions. The robotic fish is designed using a minimalistic approach including a rigid body, and robotic fish fins. A customized fish-food dropping unit is included in the robotic fish in order to achieve behavioral changes in the fish whereby the robotic fish gains the attention of the fish while guiding them to change their behavior. The effectiveness of these maneuvers is evaluated in the study. The robotic fish is capable of identifying fish in the water, accurately identifying the fish swarm patterns, and changing the behavior of the fish swarm patterns through synchronous pre-defined moves.

## 3. Design and Methodology

### 3.1. Design of the Robotic Fish

**Figure 1:**
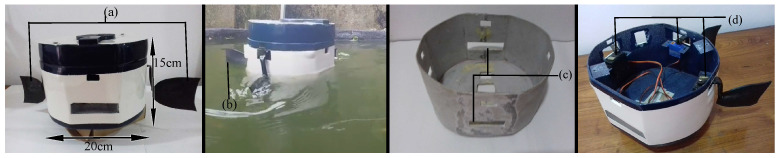
Basic appearance of the robot. From left to right. (1) Final Design of the robotic fish. (**a**) Robot fish fins. (2) Robot floating in a fish tank, (**b**) Tail of the robot. (3,4) Rigid robotic fish body and internal structure of the robotic fish. (**c**) Two acrylic boards are used for the front, and bottom to place cameras inside the robot. Front: IR camera to capture the fish behaviors around the water surface. Bottom: GoPro camera for capturing fish behaviors at the bottom area of the fish tank. (**d**) Servo motor placements for robot fins.

Research on social robots incorporates a minimalistic approach when designing the exterior to obtain optimum human–robot interaction [37]. The most common approach to creating robotic fish typically uses a slim body shape with a multi-jointed body structure [3]. The body displacement of fish categorizes fish into five main categories: Anguilliform, Subcarangiform, Carangiforrm, Thunniform, and Ostraciiform [13]. Our approach is to develop a model that imitates the Ostraciiform motion; thus, unlike the other methods involving carangiform and sub-carangiform locomotion, the robotic fish generates thrusts using only its caudal fin [38,39]. In designing the robotic body, the space and strength required to mount the hardware devices must be considered, resulting in a round-shaped rigid body design with an oscillating tail that is 20 cm long and 15 cm tall. The approach of this study suggests a minimalistic design without affecting its dynamic model while encouraging interaction with humans. Figure 1 shows the implemented design of the robot that was built.

For the hardware implementation, four principal parts were considered.

#### 3.1.1. Rigid Body

As in Figure 1, the exterior structure of the robotic fish consists of two parts: body and fish fins. The body structure was constructed by supporting the weight of the hardware mounted on the fish while maintaining buoyancy. The main concern in being thorough in the body’s construction was to guarantee the waterproofing so the hardware would function correctly. A three-dimensional (3D) model for the top cover was designed with Auto desk fusion 360 and printed with a 3D Printer using Acrylonitrile Butadiene Styrene (ABS) as the printing material with a 2 mm thickness and a solidity ratio of 90, following the same procedure to smooth out and waterproof the surface.

#### 3.1.2. Robotic Fish Fins

The robotic fish consists of a caudal fin and two equivalent pectoral fins, where previous studies propose rectangular, trapezoidal, and bio-inspired fin geometries considering the higher body mass of the robotic fish, as per a study by Matteo et al. [21]. The robotic tail was constructed with flexible material to obtain a smooth undulation instead of a rigid tail. As in Figure 1, fins built with plastic and 2 mm thick lightweight rubber sheets were attached to the bent copper arms connected directly to the servo motors inside the robot body.

#### 3.1.3. Internal Structure

**Figure 2:**
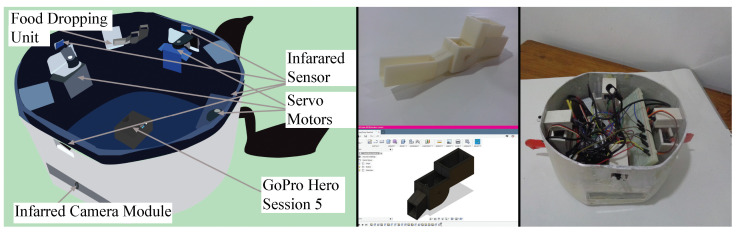
From left to right. (**Left**) Illustration of the 3D model of the robot with sensors. (**Middle**) 3D printed fish dropping unit and its 3D design. (**Right**) Sensors are put into the robot in the initial stage of development.

As in Figure 1 and Figure 2, three servo motors were balanced on the left, right, and back of the base, and a screw was fixed to the metallic frame of the body to control the fins. The Raspberry Pi camera was mounted vertically at the front area of the body in a specially printed 3D casing to capture the behavior of the fish in front of the robotic fish.

#### 3.1.4. Food Dropping Unit

Activating the aquatic environment by providing fish food is one of the main objectives of this study. The custom-made food dropping unit consists of a food container, a wheel, and an outer canal, as shown in Figure 2. The Cyprinus carpio (common carp) fish used in this research are bottom-feed fish types [40]. In order to detect fish, it is important to keep fish near the bottom of the fish tank. Hence, dried hand-made fish food is selected for simplicity of fish detecting and tracking purposes. Once the fish food was loaded into the container by rotating the wheel, it discharges the food through the channel to the fish tank as in Figure 2.

This functionality starts after receiving the signal from a proximity sensor which is located outside the aquatic environment. Fish are provided food in the water for a predefined time period. The time for each fish drop pattern is variable. Providing fish food at regular intervals may affect the water quality. Hence, according to the fish sample size of our study, 5 is the maximum number of times food drop behavior can occur per day.

### 3.2. Hardware Architecture of the System

#### Internal Structure

**Figure 3:**
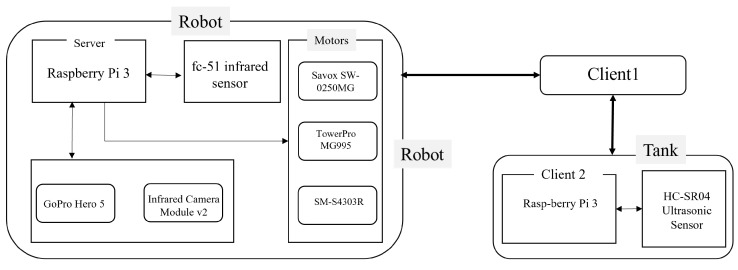
Hardware architecture of the developed robotic system with sensors.

In this study, two Raspberry Pi Model B micro-controllers, each equipped with a 2.4 GHz 802.11n wireless network, were utilized. One Raspberry Pi (referred to as the “server”) was placed inside the robot’s rigid body and was responsible for controlling the servo motors, cameras, and sensors. The video frames captured by two cameras were transmitted to a computer (referred to as “client1”) and received the necessary control signals via web sockets. The second Raspberry Pi (referred to as “client2”) was installed outside of the aquatic environment and was equipped with a proximity sensor system to detect human presence. Figure 3 displays the schematic diagram of the hardware for the robotic system.

Three types of servo motors were used in this study. A Savox SW-0250MG waterproof servo motor was used to control the caudal fin and had a speed of 60 degrees per 0.11 s and generated a torque of 5 kg per 1 cm. However, the robot required a more powerful torque-generating servo motor with momentum towards the front direction, so the TowerPro MG995 servo motors were used to control the pectoral fins. These servo motors had a speed of 60 degrees per 0.13 s and generated a torque of 12 kg per cm. In addition, a custom-made food dropper unit was built using an SM-S4303R 360-degree rotation servo motor, which operated at a speed of 60 rounds per minute and generated a torque of 4.8 kg per cm.

The GoPro Hero 5 Session, a waterproof camera capable of operating in depths of up to 30 feet and taking high-resolution photos, was used as the bottom camera in this study. It was used to collect images for training the neural network and to capture real-time images while the robot was in operation. An IR camera module, v2 (Pi NoIR), was used as the front camera of the robotic fish. This camera was used to detect and track fish swarms, allowing the robot to maneuver towards them. The camera was fixed to the server Raspberry Pi and controlled by the main program of the robotic fish. Additionally, the camera was able to capture fish that were swimming and behaving closer to the surface of the water.

An FC-51 IR Obstacle Avoidance sensor module was used in this study. It consisted of a pair of IR transmitters, an IR emitting tube, and a receiver tube. The module also included a potentiometer knob to adjust the detection distance. In this study, four sensors of this module were used to detect fish tank surfaces and other obstacles in the aquatic environment. The sensors were named front, rear, left, and right, respectively, and were horizontally located 4 cm below the robotic fish’s roof. The left and right sensor detection distance was set to 10 cm, whereas the front and rear sensor obstacle detection distances were set to 20 cm and 25 cm, respectively. The output port of the sensors was directly connected to the Raspberry Pi microcontroller. The HC-SR04 Ultrasonic Sensor was used to detect the presence of humans in the aquatic environment. This module had a detection range of 2 cm to 400 cm, allowing the robotic fish to respond in a timely manner by changing its behavior. Four sensors were installed outside the fish tank and all sensors were connected to the client2 Raspberry Pi and controlled by it.

### 3.3. Experimental Setup

**Figure 4:**
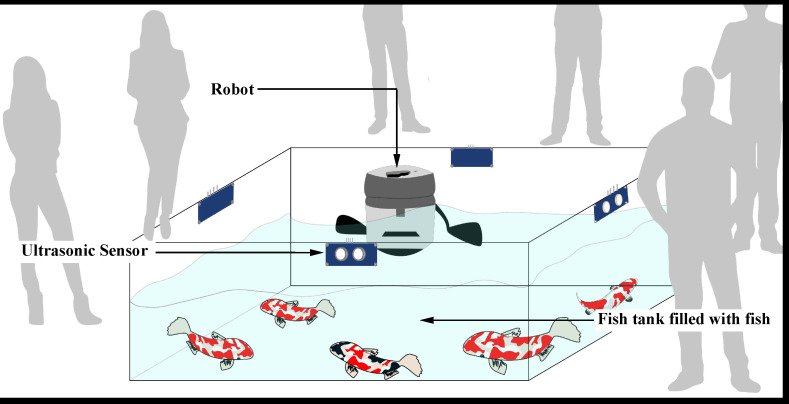
An illustration of the experimental setup used in the research, which includes ultrasonic sensors for detecting human presence.

The authors of this study, one of whom is an enthusiast for Cyprinus carpio fish during his leisure activities, created an experimental setup in a home garden. An aquatic environment measuring 240 cm × 120 cm × 90 cm was constructed to detect patterns and develop algorithms. The tank was filled with fresh water and 10 Cyprinus carpio fish of different sizes were placed inside. Ultrasonic sensors were installed around the fish tank. The fish were introduced to the environment and the robotic fish was deployed. The fish were given time to adapt to the new environment and to start performing their natural swarm behaviors. Data collection for the fish-detecting algorithm was performed using the GoPro camera located at the bottom of the robot. Additionally, a web camera was set up on top of the tank to observe the behavior of the robot and the fish. Figure 4 illustrates the experimental setup used in this research.

### 3.4. Software Implementation

**Figure 5:**
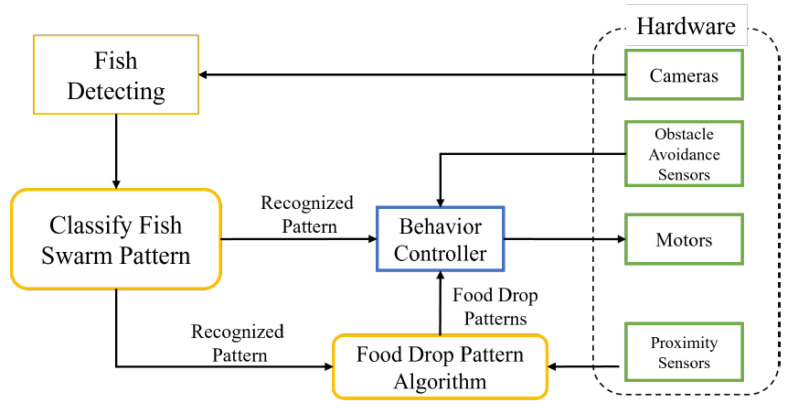
The simplified software architecture of the robotic fish is illustrated with the use of color coding, where sensors are represented in green, algorithms in yellow, and robot behaviors in blue.

Figure 5 illustrates the control flow of the robot. All video frames captured by the two cameras were sent to the fish detection algorithm to detect the position of fish in each frame. In order to ensure that the video frames are transmitted in the correct order, Transmission Control Protocol (TCP) sockets were used to stream the video frames between the server and the client.

#### 3.4.1. Fish Detecting Algorithm

**Figure 6:**
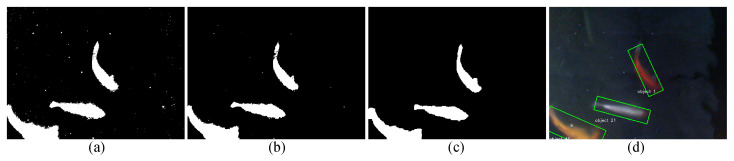
The fish detection process is composed of four stages, as illustrated: (**a**) input image is closed and then opened, (**b**) the closed and opened image is eroded, (**c**) the eroded image is closed, (**d**) final output image with fish detection is obtained.

Detecting moving fish using moving robots brings more complexity to the system. Removing the background noise of the video frames taken from the GoPro camera, caused by the surrounding fluid and other environmental obstacles makes detecting fish more challenging. We incorporated background subtraction with the moving average method [41]. Let *BS* be the output image after applying background subtraction, and *MA* be the output image of the moving average algorithm. Following Equation (1), which describes background subtraction,

(1) $$\begin{array}{ccc} ∥l\left(x^{\prime },y^{\prime },t\right)-l\left(x^{\prime },y^{\prime },t-1\right)∥ & > & Th. \end{array}$$

where $x^{\prime }$ and $y^{\prime }$ are the pixel position, *t* denotes the time, *l* denotes the photo label, and *Th* represents the threshold value. We combined *BS* and *MA* using the bitwise “And” operation and applied 15 times of image closing, 15 instances of image opening, 2 instances of image eroding, and 10 instances of image closing, respectively, for the noise cancellation. Furthermore, filters are used for both closing (with a kernel size of 2 × 2) and opening (with a kernel size of 1 × 1) operations, and then the center points of each moving fish were calculated. Figure 6 shows the sample output that is obtained from the fish detection.

#### 3.4.2. Classify Fish Swarm Patterns

**Figure 7:**
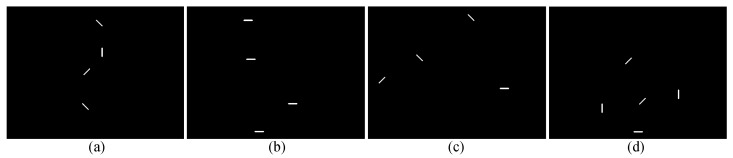
The selected fish swarm patterns in the study are illustrated: (**a**) Fish Schooling-Following, (**b**) Fish Schooling-Parallel, (**c**) Shoal, and (**d**) Fish Schooling-Tornado patterns.

The limited field of view of the robotic fish makes it impossible to classify all fish swarm behaviors. Therefore, four patterns were defined as the corpus: Fish Schooling-Following (pattern 1), Fish Schooling-Parallel (pattern 2), Shoal (pattern 3), and Fish Schooling-Tornado (pattern 4). A Convolutional Neural Network (CNN) was used to classify fish swarm behavior. Images taken from the camera set up on top of the tank were used for this purpose. A Python program in OpenCV was created that captures fish positions and saves frames every minute. The program first generates a blank image and, then, depending on the fish’s moving direction, it places a marker on the empty image. Figure 7 illustrates the four fish swarm patterns selected for the study and the outcome image of the detection algorithm.

The GoPro camera collected and labeled 1200 images according to fish swarm patterns, which was not a sufficient training set. To increase the dataset, image augmentation techniques such as image rotation, translation, flipping, and cropping were used to bring the dataset up to 1620 images. The CNN classifier was used, the first layer of which is the convolution layer, which extracts features from the input images and convolves the features to the feature map, followed by applying the Rectified Linear Units (ReLU) activation function, which is used after every convolution operation, and then followed by the pooling layer. Equation (2) describes the ReLU activation function,

(2) $$\begin{array}{ccc} f(i) & = & max(0,i), \end{array}$$

where *i* denotes the input for a particular neuron. In this study, two sets of layers were used before the fully connected layers. In the first set of layers, the convolution layer takes 224 × 224 × 3 images as input and learns 32 convolution filters with a size of 3 × 3. Then, the ReLU activation function followed by a max-pooling layer with a 2 × 2 stride window was applied. The second set of layers starts with a convolution layer with 64 convolution filters of size 3 × 3 to increase the depth of the network. Then, the classifier includes a flattened layer that flattens the previous layer’s output to a single vector array. We used two fully connected layers (Dense layers); the first fully connected layer contains 1024 nodes which take a single array provided by the flattened layer as input; then, the ReLU activation function is applied. Since only four selected fish swarm behaviors were considered, the last fully connected layer contains only four output nodes followed by a softmax activation function. Furthermore, the Adam optimizer was used to optimize the classifier. Since this was a classification problem, categorical cross-entropy was used as the loss function.

### 3.5. Dynamic Model of Robotic Fish

**Figure 8:**
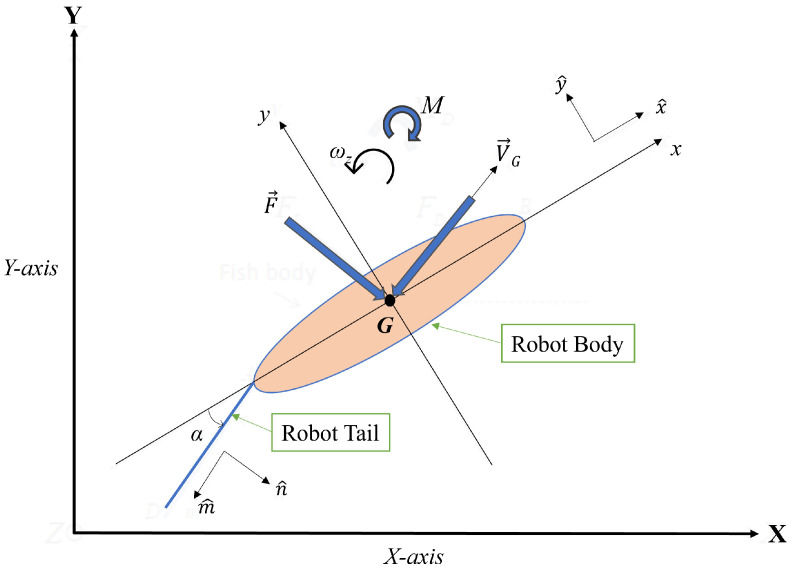
Top view of robotic fish in Cartesian coordinate system undergoing planar motion.

Since the rigid body and oscillating tail are the two main parts of the robotic fish, hydrodynamic forces are generated as opposed to the robot by the robotic fish actuating tail, described by Lighthill’s large-amplitude elongated-body theory [42]. The dynamic model used in our study combines both rigid body dynamics and Lighthill’s theory [43]. Figure 8 shows the top view of the robotic fish while undergoing planar motion.

Suppose [X, Y, Z] denote the Cartesian coordinates, and [x, y, z] represent the body-fixed coordinates concerning to robotic fish. $\hat{x}$, $\hat{y}$, $\hat{z}$ are unit vectors along x, y, z, respectively. Throughout this study, we assume the centroid and center of gravity coincide at point G and that the robotic fishtail only oscillates in the xy-plane. Further, we assume that the robotic fish’s rigid body is symmetric to the xz-plane. Moreover, $\hat{m}$ denotes a unit vector parallel to the tail and $\hat{n}$ denotes a unit vector perpendicular to the tail. The velocity at G and angular velocity can be expressed as, ${\vec{V}}_{G}={\left[V_{G}x,V_{G}y,V_{G}z\right]}^{T}$, $\vec{\omega }={\left[\omega_{x},\omega_{y},\omega_{z}\right]}^{T}$, respectively. The tail defection angle with respect to negative x-axis denoted by θ and α denotes angle between X-axis and x-axis. Using Kirchhoff’s equations of motion,

(3) $$\begin{array}{ccc} \dot{\vec{P}} & = & \vec{P}\times \vec{\omega }+\vec{F}, \end{array}$$

(4) $$\begin{array}{ccc} \dot{\vec{H}} & = & \vec{H}\times \vec{\omega }+\vec{P}\times \vec{V_{G}}+\vec{M}, \end{array}$$

where $\vec{P}$ denotes linear momentum and $\vec{H}$ represents angular momentum of the rigid body [44,45]. $\vec{F}={\left[F_{x},F_{y},F_{z}\right]}^{T}$, $\vec{M}={\left[M_{x},M_{y},M_{z}\right]}^{T}$ indicate external forces and moments about *G*, respectively. Hydrodynamic forces exerted on the tail were evaluated by Lighthill’s large-amplitude elongated-body theory [43]. For simplicity, we take $V_{Gx}$, $V_{Gy}$, $\omega_{z}$ as *u*, *v*, *r*, respectively,

(5) $$\begin{array}{ccc} \left(m_{b}-X_{\dot{u}}\right)\dot{u} & = & \left(m_{b}-Y_{\dot{v}}\right)vr+F_{x}, \end{array}$$

(6) $$\begin{array}{ccc} \left(m_{b}-Y_{\dot{v}}\right)\dot{v} & = & \left(m_{b}-X_{\dot{u}}\right)ur+F_{y}, \end{array}$$

(7) $$\begin{array}{ccc} \left(J_{bz}-N_{\dot{r}}\right)\dot{r} & = & \left(Y_{\dot{v}}-X_{\dot{u}}\right)uv+M_{z}, \end{array}$$

where $J_{bz}$ is the inertia and $m_{b}$ is the mass of the robotic fish body, including pectoral fins. Finally, $X_{\dot{u}}$, $Y_{\dot{v}}$ and $N_{\dot{r}}$ represent hydrodynamic derivatives.

**Figure 9:**
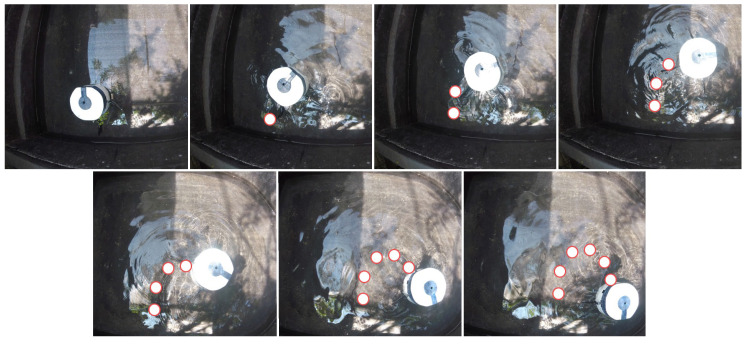
Video Sequences of C-Shape turning maneuver of the robotic fish.

**Figure 10:**
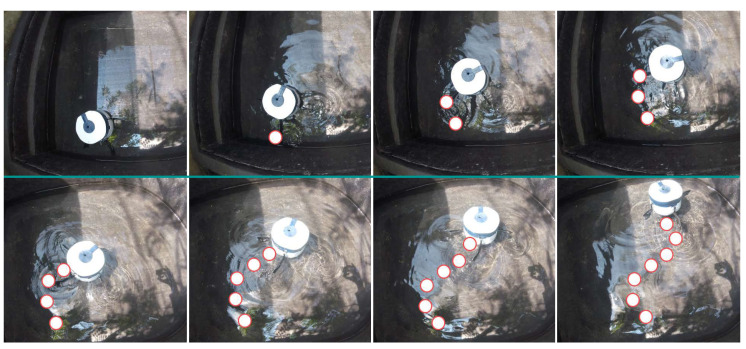
Video Sequences of S-Shape turning maneuver of the robotic fish.

We designed the robot’s maneuvers to suit its minimalistic design; therefore, vertical plane maneuvers, such as up/down swimming motions, acceleration, deceleration, and hovering, were not included in the corpus. To mimic fish-like swimming behaviors, carangiform and sub-carangiform robotic fish were developed with C-shape sharp turns, and S-shape turns [38]. Figure 9 and Figure 10 show video sequences of the two main rotating patterns. The white object in the pictures is the robot.

### 3.6. Data Collection

**Figure 11:**
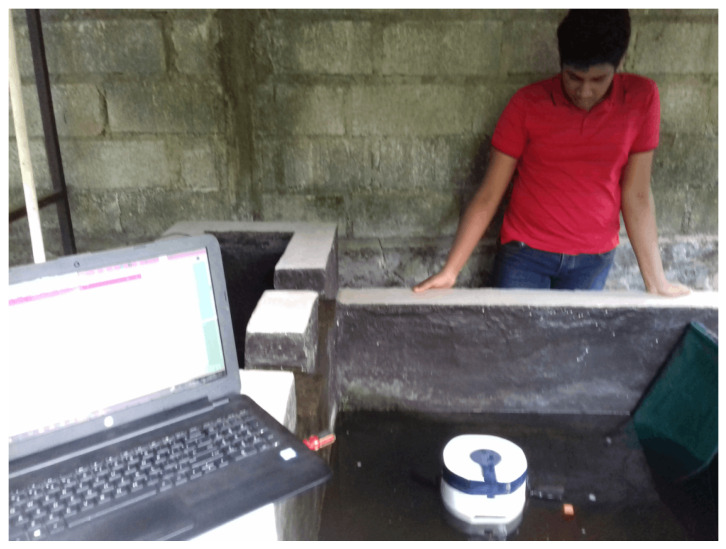
Experimental Setup with a participant.

The experiment was conducted with 15 participants of varied ages (18–57), sex, and fish-rearing experience. The participants were instructed to stand in front of the fish tank, as shown in Figure 11, and observe the behavior of the fish.

Participants were given a questionnaire consisting of two parts, one before the experiment and the other after the investigation. For improved visibility and analysis purposes, questions were categorized into five sections.

- Part 1: information about the participant (5 questions);
- Part 2: animacy of the robotic fish (3 questions);
- Part 3: design of the robotic fish (3 questions);
- Part 4: the effectiveness of robotic fish behaviors (3 questions);
- Part 5: the overall impression of robotic fish (3 questions).

The first part of the questionnaire contained questions to identify the participant. Important quantitative and qualitative data such as sex, age, and prior fish-rearing experience were included in this section, which were utilized in the data analysis section. The second part of the questionnaire included questions that evaluated the animacy level of the robot. This part aimed to measure the participant’s first impressions towards the robot and whether they perceived it as a real fish. Additionally, it aimed to measure whether the robotic fish had a deviation from the real fish maneuvers or not. The third part of the questionnaire contained questions that evaluated the physical appearance of the robot. This part aimed to measure user acceptance of the robotic fish and also included the participant’s impressions towards the robot, as well as whether they preferred the minimalistic and futuristic design of the robotic fish. The fourth part of the questionnaire contained questions to measure the effectiveness of the robot fish’s behaviors, including each maneuver. This part focused on whether the robotic fish was able to change the whole fish swarm patterns by its set of maneuvers. Furthermore, user feedback was used in this part to evaluate whether the changed swarm patterns were suitable for the situation. The final part of the questionnaire focused on the participant’s overall impression and satisfaction with the robotic fish. This part included an overall impression of the robot’s behaviors and whether the robotic fish was able to enhance the interaction while keeping the aquatic environment lively and reducing the stress levels of the participants.

## 4. Results and Discussion

### 4.1. Fish Detection

When it comes to previous works, few researchers have examined fish detection in the past few years. Almero et al. tried to detect common carp fish in a tank using a small data size [46]. Rekha et al. used CNN for fish detection and had an accuracy rate of 90%. However, they used images of the camera feeds from fishing boats, and the dataset was populated with fish outside the water [47]. Research by Christensen et al. indicated a low fish detection accuracy due to the complexity and clearness of the selected dataset images [48]. Han et al. tried to detect fish shoals using a top view camera placed on the top of the tank [49]. The accuracy changed according to the different batch sizes they used. Table 1 shows the summary of the previous works.

To analyze the accuracy of the fish detection algorithm, a random sample of 200 images was chosen from a population of 1620 images using a random sampling method. As mentioned above, the confusion matrix generated by manually separating the sample images into categories was used in analyzing the algorithm’s accuracy. The confusion matrix is shown in Table 2, and the confusion matrix measurements are shown in Table 3.

**Figure 12:**
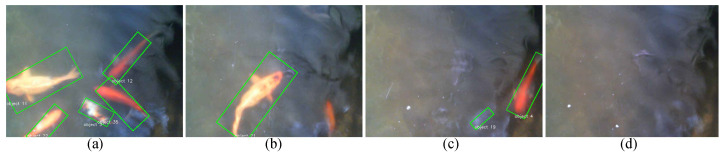
The categories of each type. From left to right: (**a**) True Positive (TP), (**b**) False Negative (FN), (**c**) False Positive (FP), (**d**) True Negative (TN).

As shown in Table 2, the images were manually separated into four categories to generate a confusion matrix responding to the fish detection algorithm. Figure 12 shows the images of the four categories.

The calculated value for the accuracy of the fish-detecting algorithm was 78%. Swirling vortexes and moving air bubbles from bottom to top may be the reason behind the false positive score, and poor water quality and lightning issues may be the reason behind the false-negative score. Therefore, the impact of these factors affected the accuracy of the fish detection algorithms.

### 4.2. Fish Swarm Pattern Recognition

Thirty percent of the dataset was randomly extracted as the test set (a total of 250 images), and CNN was trained for 25 epochs. Table 4 below shows the confusion matrix for all classes (Patterns) without normalization.

Compared to other machine learning models, the accuracy of the fish swarm-pattern recognition model is comparatively low [50]. The dataset’s quality may be the main reason behind this since most of the images used for training and for testing were from two completely different sessions. The accuracy of this model is 77%. There were studies with better accuracy than the model created in this study. However, those models required high-end computing resources and were not available for public use. Additionally, those models took considerable time to produce outcomes, while this model’s average time to create an outcome was 5–10 s per image.

### 4.3. Food Drop Patterns

In this study, robotic fish performed four contrasting food drop patterns to enhance the aquatic environment’s liveliness by improving the fish’s attraction. Data captured for the fish-food drop patterns and the classified fish swarm patterns were analyzed. Table 5 contains the number of results of fish-food drop patterns that produced the expected fish swarm patterns.

**Figure 13:**
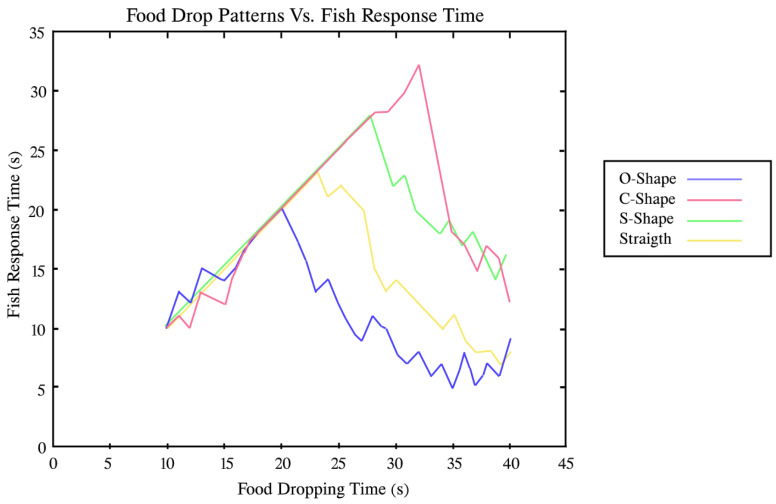
Fish responses for each of the food drop patterns.

According to the results, the C-shape food pattern had a higher percentage (55.6%) with the fish Schooling-Parallel pattern (Pattern 2). In contrast, all patterns have similar percentages to the fish Schooling-Following pattern (pattern 1). Furthermore, the O-shape and Straight food patterns were 40.9% and 36.4%, percentages, respectively, with the Shoal pattern (pattern 3). Compared with the Fish Schooling Parallel pattern, the C-shape food pattern and O-shape food pattern were 35.3% and 47.1%, respectively. Fish response to particular fish-food drop patterns varied according to the time. Figure 13 shows fish responses for C-shape and O-shape patterns and that the fish lost their interest at 32 and 19 s, respectively. Table 6 contains the maximum time (in seconds) for each fish food pattern along with fish interest.

After analyzing the results on fish-food drop patterns and changing fish swarm behaviors, pattern 1 behavior responded to all the food drop patterns. Pattern 2 behavior of fish responded mostly to C-shape and straight food drop patterns. Furthermore, pattern 3 fish behavior reacted only to the O-shape food drop pattern, while pattern 4 fish behavior responded to both O-shape and C-shape food drop patterns.

### 4.4. Data Collected through the Questionnaire

Table 7 shows the summarized feedback from the questionnaire for the robotic fish.

**Figure 14:**
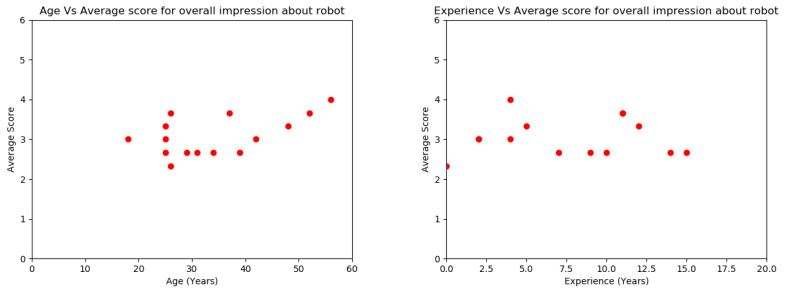
(**Left**) Relationship with age and the average score for overall impression. (**Right**) Relationship with experience and the average score for overall impression.

The animacy, design of the robotic fish, and overall satisfaction achieved better user feedback compared to the effectiveness of robotic behaviors, which have achieved moderate user feedback. Since all the questions from parts 2–5 are on the same Likert scale, the average value of the output was obtained in each of the sections. As shown in Figure 14, when the age increases, the overall impression of robotic fish is also increasing. As in Figure 14, it was also found that there was no significant relationship between the amount of experience and the average score for the overall impression of robotic fish. According to the data collected from participants as feedback, some of them suggested that their moods and stress levels were not the same throughout the experimental time. Some suggested that more fish drop patterns could generate different fish swarm patterns. Hence, additional fish drop patterns should be used to change the fish swarm behaviors.

## 5. Conclusions and Future Works

The developed robotic fish could detect the fish in the water, and identify their swarm pattern with a significant accuracy of 78% and 77%, respectively. The robotic fish was able to alter fish swarm behavior through synchronous pre-defined maneuvers where the highest percentage of 55.6% was shown for fish Schooling-Parallel swarm pattern for the C-shape fish-food drop pattern. At the initial stage, the robotic maneuvers were not smooth, and the fish-dropping mechanism did not perform to expectations; however, the revised controlling algorithm objective was achieved, producing improved maneuvers. According to the collected data using subjective feedback from the 15 participants, their stress was comparatively enhanced mainly through the generated fish swarm patterns by the robotic fish. A larger proportion of participants agreed that the overall impression of the robotic fish was positive. Assessing the results obtained, it was evident that the robotic fish was capable of detecting fish, identifying fish swarm patterns, and ultimately inducing swarm behavior in the fish which successfully altered the swarm patterns of the fish.

We encountered difficulty obtaining clear pictures using the camera modules we utilized to detect fish underwater, and at one point, the GoPro camera suffered water damage. It is recommended to use more advanced camera sensors in future projects. The food drop pattern was determined by a simple random assignment algorithm, which could be improved by implementing a learning algorithm such as Q-learning to enable the robot to predict the optimal drop pattern for maximum interaction. Additionally, the study was limited to a single fish species and a small aquatic environment. The study could be further strengthened by including multiple fish species and a larger marine environment.

## Figures and Tables

**Table 1:** Summary of previous research.

Author	Data Size	Training Testing Ratio	No. of Classes	No. of Layers	Accuracy
Almero et al. [46]	369	2:1	2	4	79.00%
Rekha et al. [47]	16,000	8:2	8	15	90.00%
Christensen et al. [48]	13,124	NA	3	6	66.7%
Hanet et al. [49]	600	4:1	6	4	82%

**Table 2:** Confusion matrix for fish detection.

n = 200	Detected Yes	Detected No
Fish Present Yes	TP = 121	FN = 27
Fish Present No	FP = 17	TN = 35

**Table 3:** Confusion matrix measurements for fish detection.

Measure	Derivation	Value
Accuracy	ACC = (TP + TN)/(P + N)	78.00%
Sensitivity	TPR = TP/(TP + FN)	81.75%
Specificity	SPC = TN/(FP + TN)	67.30%
Precision	PPV = TP/(TP + FP)	87.68%

**Table 4:** Confusion matrix for pattern recognition.

	Classified	Pattern 1	Pattern 2	Pattern 3	Pattern 4
Actual					
Pattern 1		49	8	15	0
Pattern 2		2	73	6	13
Pattern 3		8	7	42	0
Pattern 4		0	8	1	18

**Table 5:** Relationship between fish swarm patterns and selected fish-food drop patterns.

	Food Drop Patterns	S-Shape	C-Shape	O-Shape	Straight
Fish Swarm Patterns					
Fish Schooling-Following		26.7%	23.3%	20.0%	30.0%
Fish Schooling-Parallel		11.1%	55.6%	11.1%	22.2%
Shoal		13.6%	9.1%	40.9%	36.4%
Fish Schooling-Tornado		5.9%	35.3%	47.1%	11.8%

**Table 6:** Maximum fish response time for each fish food pattern.

Food Pattern Name	Maximum Fish Response Time (s)
Straight pattern	22
S-shape pattern	28
C-shape pattern	32
O-shape pattern	19

**Table 7:** Summarized results of the questionnaire.

User Feedback	Strongly Agree	Agree	Not Sure	Disagree	Strongly Disagree
Part 2	20.0%	48.9%	31.1%	0.0%	0.0%
Part 3	51.1%	37.8%	11.1%	0.0%	0.0%
Part 4	22.2%	26.7%	33.3%	17.8%	0.0%
Part 5	35.6%	42.2%	22.2%	0.0%	0.0%

## Data Availability

Not applicable.
